# Heritable methylation marks associated with prostate cancer risk

**DOI:** 10.1007/s10689-022-00325-w

**Published:** 2023-01-28

**Authors:** James G. Dowty, Chenglong Yu, Mahnaz Hosseinpour, Jihoon Eric Joo, Ee Ming Wong, Tu Nguyen-Dumont, Joseph Rosenbluh, Graham G. Giles, Roger L. Milne, Robert J. MacInnis, Pierre-Antoine Dugué, Melissa C. Southey

**Affiliations:** 1 Centre for Epidemiology and Biostatistics, Melbourne School of Population and Global Health, The University of Melbourne, 3010 Parkville, VIC Australia; 2 Precision Medicine, School of Clinical Sciences at Monash Health, Monash University, 3168 Clayton, VIC Australia; 3 Cancer Epidemiology Division, Cancer Council Victoria, 3004 Melbourne, VIC Australia; 4 Department of Clinical Pathology, Melbourne Medical School, The University of Melbourne, 3010 Parkville, VIC Australia; 5 Cancer Research Program, Department of Biochemistry and Molecular Biology, Biomedicine Discovery Institute, Monash University, 3800 Clayton, VIC Australia

**Keywords:** DNA methylation, Trans-generational inheritance, Heritable methylation marks, Prostate cancer risk, Familial prostate cancer, Aggressive prostate cancer

## Abstract

DNA methylation marks that are inherited from parents to offspring are known to play a role in cancer risk and could explain part of the familial risk for cancer. We therefore conducted a genome-wide search for heritable methylation marks associated with prostate cancer risk. Peripheral blood DNA methylation was measured for 133 of the 469 members of 25 multiple-case prostate cancer families, using the EPIC array. We used these families to systematically search the genome for methylation marks with Mendelian patterns of inheritance, then we tested the 1,000 most heritable marks for association with prostate cancer risk. After correcting for multiple testing, 41 heritable methylation marks were associated with prostate cancer risk. Separate analyses, based on 869 incident cases and 869 controls from a prospective cohort study, showed that 9 of these marks near the metastable epiallele VTRNA2-1 were also nominally associated with aggressive prostate cancer risk in the population.

**Supplementary Information:**

The online version contains supplementary material available at 10.1007/s10689-022-00325-w.

## Introduction

Most DNA methylation marks are erased before or soon after conception, however methylation marks are known to be effectively inherited from parents to offspring in rare cases, e.g. near *MLH1* and *MSH2* [1]. Such heritable methylation marks can be caused by genetic variation, in which case the relevant genetic loci are called methylation quantitative trait loci (mQTLs), or due to non-genetic causes, in which case the methylation mark is called an epimutation [2].

Some epimutations, and most methylation marks that are strongly associated with an mQTL, are systemic (i.e. they affect all tissues) [3], which makes them easily detectable in blood and enables an assessment in blood to predict cancer risk in other tissues. Epimutations can mimic germline pathogenic variation, and their contribution to cancer predisposition may have been underestimated because previous studies have mainly used candidate-gene approaches, though some genome-wide searches for heritable methylation marks have more recently been conducted, e.g. [3, 4].

One of the most important risk factors for prostate cancer is having a family history of the disease but less than half of the familial risk for prostate cancer is explained by the currently identified genetic risk factors. This is despite modern genomic studies of prostate cancer risk being based on tens of thousands of cases, suggesting that many heritable risk factors for prostate cancer might exist that are not genetic. We therefore conducted a systematic, genome-wide search for heritable methylation marks associated with prostate cancer risk, and assessed the extent to which their variation was explained by common genetic variants.

## Methods

Our study was based on previously reported methods [3, 5, 6] and was conducted in two phases, a family-based phase and a population-based phase (see Supplementary Methods for more details).

The family-based phase was based on 25 multiple-case prostate cancer families drawn from the Australian Prostate Cancer Family Study (APCFS​) [7]. Peripheral blood DNA methylation was measured for 133 of the 469 family members using the Infinium methylation EPIC array. To identify heritable DNA methylation marks (whether epimutations or mQTLs), we calculated a measure of heritability, $\varDelta l$, for each methylation mark, with high values of $\varDelta l$ corresponding to Mendelian patterns of inheritance within the families [3]. Methylation marks on sex chromosomes or within 10 base pairs of a known SNP were excluded.

Because we were interested in causes of familial prostate cancer, we selected only the 1,000 most heritable methylation marks. For each of these, we calculated the probability that each family member carries a hypothetical genetic variant causing aberrant methylation at the mark, based on M-values and family structure but not ages or affected statuses. We tested these carrier probabilities for association with prostate cancer using Cox proportional hazards survival models. We accounted for multiple testing (for 1,000 tests) using the Bonferroni p-value threshold of 0.05/1000. Risk estimates from the family-based phase are biased by ascertainment so are not presented, though p-values are valid because the test statistic is not affected by ascertainment under the null hypothesis.

The population-based phase was based on unrelated individuals recruited irrespective of family history to the Melbourne Collaborative Cohort Study (MCCS) [8, 9]. Peripheral blood DNA methylation was measured in 869 incident cases (including 430 aggressive cases) and matched controls (matched on year of birth, year of blood draw, country of birth, and sample type) using the HM450 array, as described previously [8, 9]. This data was used to further investigate the marks from the family-based phase that are heritable, associated with prostate cancer risk, and common to the EPIC and HM450 arrays (the two arrays used in the two phases). These marks were tested for association with prostate cancer using conditional logistic regression adjusted for body-mass index, tobacco smoking, alcohol consumption, age at blood draw and estimated blood cell composition. A genome-wide search for mQTLs was also conducted for these methylation marks, using 4,307 unrelated MCCS participants genotyped on the OncoArray-500 K BeadChip [6]. Sites near *VTRNA2-1* have bimodal distributions so, as a sensitivity analysis, we also dichotomised the methylation values of these sites and estimated their associations with prostate cancer using conditional logistic regression, as in the main analyses, above.

## Results

The 1,000 most heritable methylation marks from the family-based phase are listed in Supplementary Table 1. Of these 1,000 methylation marks, 41 were associated with prostate cancer risk at the Bonferroni-corrected significance level (Table 1).

**Table 1:** The 41 heritable methylation marks associated with prostate cancer risk from the family-based phase and, for 25 of these marks that were measured in the population-based phase, their association with prostate cancer risk (overall and aggressive) in the general population

				Family-based phase		Population-based phase, associations per 1 standard deviation of M-values with					
Methylation mark	Chr.	Position (GRCh37)	Nearby gene	$\varDelta l$	p-value for association with prostate cancer	Prostate cancer ^a^		Aggressive prostate cancer ^a^		SNPs ^b^	
						OR (95% CI)	p	OR (95% CI)	p	Number of SNPs ^c^	R^2^ for the top SNP ^d^
cg06536614	5	135,416,381	*VTRNA2-1*	102.2	1E-07	0.90 (0.82-1.00)	0.05	0.85 (0.73–0.98)	0.03	2	0.0081
cg20443278	17	77,962,098	*TBC1D16*	93.0	3E-06	1.10 (0.92–1.32)	0.3	1.16 (0.90–1.51)	0.2	33	0.0174
cg26896946	5	135,416,405	*VTRNA2-1*	85.2	2E-07	0.90 (0.81–0.99)	0.03	0.84 (0.72–0.97)	0.02	0	0
cg25340688	5	135,416,398	*VTRNA2-1*	79.7	6E-08	0.91 (0.82-1.00)	0.05	0.84 (0.73–0.97)	0.02	0	0
cg00124993	5	135,416,412	*VTRNA2-1*	67.5	2E-08	0.90 (0.81–0.99)	0.03	0.83 (0.72–0.96)	0.01	0	0
cg20124410	13	107,333,224		59.0	9E-07	-	-	-	-	-	-
cg12012426	4	1,366,463	*KIAA1530*	55.7	2E-05	1.07 (0.97–1.18)	0.2	1.07 (0.93–1.22)	0.3	1578	0.6629
cg20054939	12	133,614,314	*ZNF84*	50.5	4E-05	1.10 (1.00-1.22)	0.06	1.14 (0.98–1.32)	0.1	21	0.4940
cg20004147	2	65,718,931		49.4	4E-05	-	-	-	-	-	-
cg11608150	5	135,415,948	*VTRNA2-1* ^e^	46.9	6E-07	0.91 (0.83–1.01)	0.08	0.83 (0.72–0.96)	0.01	1	0.0070
cg18797653	5	135,416,613	*VTRNA2-1*	46.7	2E-09	0.92 (0.84–1.02)	0.1	0.85 (0.74–0.99)	0.03	1	0.0073
cg14159672	1	205,819,179	*PM20D1*	44.6	5E-05	0.98 (0.88–1.08)	0.6	0.94 (0.82–1.09)	0.4	979	0.7958
cg21501207	1	162,383,000	*SH2D1B*	42.1	2E-05	-	-	-	-	-	-
cg04481923	5	135,416,205	*VTRNA2-1*	41.9	5E-07	0.91 (0.83-1.00)	0.06	0.84 (0.72–0.97)	0.02	0	0
cg09483595	5	158,878,380	*LOC285627*	41.8	5E-06	0.99 (0.90–1.09)	0.8	1.07 (0.93–1.24)	0.4	243	0.1046
cg18072778	1	148,203,924	*PPIAL4F*	41.8	5E-06	1.00 (0.90–1.10)	1	1.02 (0.88–1.19)	0.8	124	0.0215
cg05141217	8	28,491,378		41.7	1E-05	-	-	-	-	-	-
cg24503407	1	205,819,492	*PM20D1*	39.9	4E-05	0.99 (0.90–1.09)	0.9	0.96 (0.83–1.11)	0.6	1023	0.7918
cg01760119	3	101,661,382	*LOC152225*	38.6	5E-08	-	-	-	-	-	-
cg13373914	7	67,323,067		38.4	2E-05	-	-	-	-	-	-
cg07157834	1	205,819,609	*PM20D1*	38.2	3E-05	0.98 (0.89–1.09)	0.7	0.94 (0.82–1.09)	0.4	1013	0.7683
cg06478886	5	135,416,029	*VTRNA2-1* ^e^	37.2	2E-09	0.90 (0.82-1.00)	0.04	0.83 (0.71–0.95)	0.01	0	0
cg26708920	10	13,826,317	*FRMD4A*	32.1	4E-07	-	-	-	-	-	-
cg10123377	3	42,387,524		31.5	6E-06	-	-	-	-	-	-
cg17714793	1	153,538,431	*S100A2*	30.6	2E-07	-	-	-	-	-	-
cg26354017	1	205,819,088	*PM20D1*	29.3	3E-05	0.98 (0.89–1.08)	0.7	0.95 (0.82–1.10)	0.5	983	0.7728
cg07158503	5	135,415,693	*VTRNA2-1* ^e^	28.4	1E-05	0.91 (0.83–1.01)	0.07	0.85 (0.74–0.98)	0.02	1	0.0071
cg16334093	1	205,819,600	*PM20D1*	28.3	4E-05	-	-	-	-	-	-
cg14893161	1	205,819,251	*PM20D1*	27.9	4E-05	0.97 (0.88–1.07)	0.6	0.93 (0.81–1.07)	0.3	981	0.8073
cg01608070	1	157,853,274		26.7	5E-06	-	-	-	-	-	-
cg19182683	4	183,730,519		26.5	1E-05	-	-	-	-	-	-
cg17884856	20	44,334,913	*WFDC10B*	26.4	5E-05	0.93 (0.85–1.03)	0.2	0.99 (0.86–1.14)	0.9	1200	0.7064
cg02722613	4	25,162,898	*SEPSECS*	25.6	2E-06	1.04 (0.94–1.14)	0.5	0.98 (0.85–1.13)	0.8	489	0.7950
cg10829391	14	101,069,717		25.5	4E-05	-	-	-	-	-	-
cg21824770	2	243,012,163	*LINC01237*	25.5	8E-06	-	-	-	-	-	-
cg26748794	16	88,804,051	*FAM38A*	23.6	2E-05	0.93 (0.85–1.03)	0.2	0.92 (0.80–1.06)	0.2	507	0.1874
cg04546999	1	152,956,429	*SPRR1A*	23.1	3E-05	0.94 (0.85–1.04)	0.2	0.92 (0.80–1.07)	0.3	555	0.0828
cg19704288	4	1,582,181		22.9	2E-05	0.98 (0.89–1.08)	0.7	1.08 (0.94–1.24)	0.3	210	0.0893
cg14150973	19	40,950,431	*SERTAD3*	22.1	1E-05	1.03 (0.93–1.13)	0.6	1.05 (0.92–1.20)	0.5	574	0.5955
cg05841700	1	205,819,383	*PM20D1*	21.5	2E-05	-	-	-	-	-	-
cg26237810	1	200,669,214		21.2	2E-07	-	-	-	-	-	-

*Abbreviations: Chr. = chromosome; GRCh37 = Genome Reference Consortium Human Build 37;* $\varDelta l$ *= a measure of heritability, with higher values indicating more heritable methylation marks; OR = odds ratio; CI = confidence interval; p = p-value; SNP = single nucleotide polymorphism; R* ^*2*^ *= the proportion of variance explained by the most associated SNP; hyphen (-) = unavailable because the methylation mark is not present on the HM450 array*

^a^ Associations were adjusted for body mass index, tobacco smoking, alcohol consumption, age at blood draw and white blood cell composition. Results for the methylation marks near *VTRNA2-1* have been previously reported [5]

^b^ dbSNP build 151 for hg19 (GRCh37), accession date 9 December 2018

^c^ The number of SNPs associated with each methylation mark’s M-values at a significance level of p < 5E-08 from a genome-wide search for mQTLs [6]

^d^ The proportion (R^2^) of the variance of the mark’s M-values explained by the SNP with the lowest p-value. Methylation marks without any mQTLs (at the significance level p < 5E-08) were assigned an R^2^ of 0

^e^ These methylation marks were within 0.5 kb of *VTRNA2-1* but were not annotated to it

Of the 41 methylation marks from the family-based phase, 25 were included on the HM450 array and so had been measured in the population-based phase. These 25 marks were tested for association with prostate cancer, and nominally significant associations (p < 0.05) with aggressive prostate cancer were found for all 9 marks near *VTRNA2-1* (Table 1), with most remaining nominally significant after dichotomising (Supplementary Table 2), (as previously reported, based on the same datasets [5]). A genome-wide search for mQTLs showed that the marks in the *VTRNA2-1* region had either no mQTLs or few and weak mQTLs, while most of the other methylation marks were associated with a substantial number of mQTLs and a large proportion of their variance was explained by a single SNP (Table 1).

## Discussion

Our study has identified 41 methylation marks associated with familial prostate cancer, and 9 of these marks (near *VTRNA2-1*) also have nominally significant associations with aggressive prostate cancer risk in the general population. Note that we would not expect all 41 methylation marks to be associated with risk in the population, e.g. *BRCA1* is usually not detected by genome-wide association studies. Also, the magnitude of risk in the population-based and familial settings could differ greatly, due to rare mQTLs or epimutations causing changes in methylation that are many times the population standard deviation.

Nine of the 41 heritable methylation marks associated with prostate cancer risk are in the imprinted *VTRNA2-1* region, with a loss of imprinting in this region consistent with a Mendelian pattern of inheritance; see also [3]. Imprinted regions are often associated with tissue growth, and a loss of imprinting can be linked to tumorigenesis, as is well-described for the *H19/IGF2* region. *VTRNA2-1* has tumour suppressor gene properties, as it regulates cell growth via inhibition of protein kinase RNA-activated (PKR). Down regulation of *VTRNA2-1* in a variety of tumours and cancer cell lines has been well documented and associated with promoter CpG hypermethylation. As we found previously using the same datasets, *VTRNA2-1* methylation marks are associated with aggressive prostate cancer in the population [5] and are largely independent of the underlying genetic sequence [6]. We and others have described the *VTRNA2-1* locus as a metastable epiallele because the loss of imprinting in this region occurs systemically, can be modulated by the periconceptional environment and persists through adulthood [10].

Seven of the heritable methylation marks associated with prostate cancer risk are located at peptidase M20 domain containing 1 (*PM20D1*), a known methylation and expression quantitative trait locus associated with risk of Alzheimer’s disease. These and the other annotated and unannotated CpGs identified in this study require further research to understand the biological explanation for their association with heritable prostate cancer risk. Non-genetic causes of heritability could not be investigated in the current study, but it is possible that familial environmental and lifestyle factors play a role in determining DNA methylation at these loci.

Despite having a modest sample size, we were able to identify many heritable, cancer-associated methylation marks. Excluding non-heritable methylation marks before testing for association with cancer excludes many marks that cannot cause familial cancer, while retaining any that can. Our method of excluding non-heritable marks before testing for association with disease is therefore a very powerful way of enriching the candidate set of methylation marks for those that could cause familial disease. Further discussion of the methodology can be found in Joo et al., [3].

Heritable methylation marks can mimic the effects of genetic variants, so identifying them is similar in many ways to finding genetic loci that are associated with cancer. As for genetic loci, these marks can implicate new biological mechanisms and therefore shed light on the processes of prostate cancer initiation and progression. These methylation marks could also be used in risk-prediction algorithms to give more precise estimates of a person’s risk of prostate cancer, and so provide more tailored screening.

Our study has several strengths, including its use of an innovative method to identify heritable methylation marks, its method of enriching the candidate set of methylation marks for those that could cause familial disease, and its use of a cohort study to further investigate the findings from the family-based phase. The main weakness of the study was that some of the marks from the family-based phase could not be investigated in the population-based phase, due to the use of different arrays in the two phases. Our modest sample size is also a weakness, though this is offset by the enrichment step described above.

In summary, our study has identified 41 heritable methylation marks that are associated with prostate cancer risk in the context of multiple-case families, with 9 of these marks near *VTRNA2-1* likely to be associated with aggressive prostate cancer in the general population.

## Electronic supplementary material

Below is the link to the electronic supplementary material.

Supplementary Material 1

Supplementary Material 2

Supplementary Material 3

## Data Availability

The datasets used and/or analysed during the current study are available from the corresponding author on reasonable request.
