# Supplementary material for: Heritable methylation marks associated with prostate cancer risk
Source: Fam Cancer. 2023 Jan 28;22(3):313–7. doi: 10.1007/s10689-022-00325-w (PMC10275808; doi:10.1007/s10689-022-00325-w)
Supplement: Supplementary file 3 — Supplementary Material 3 [file 10689_2022_325_MOESM3_ESM.docx]

# Supplementary Methods

Our study was based on previously reported methods [1-3] and was conducted in two phases, a family-based phase and a population-based phase.

The family-based phase was based on 25 multiple-case prostate cancer families drawn from the Australian Prostate Cancer Family Study (APCFS​) [4]. Peripheral blood DNA methylation was measured for 133 of the 469 family members using the Infinium HumanMethylationEPIC array. Regarding these 133 people: they were chosen because they had previously provided a blood sample; their ages (in years) had a range of 24 to 80, a median of 57, and an interquartile range of 20; they were all Caucasian; they comprised (on average) 5.3 of the 18.8 members in each family, and were scattered fairly randomly throughout their multigenerational families; they consisted of 51 prostate cancer cases, 34 unaffected males and 48 females (note that females can be informative about the heritability of methylation marks).

All bioinformatics processing was performed in R programming software (v.3.3.2). Raw intensity values were imported and processed using the *minfi* Bioconductor package [5], using the default settings. The data was normalised using the *noob* background correction and the *functional* normalisation methods [6], which were implemented in *minfi*. As in our previous study [1], individual CpG probes with detection p-values greater than 0.05 were removed from the analysis. Beta and M-values were derived using the *getBeta* and *getM* functions within *minfi*. Methylation marks on sex chromosomes or within 10 base pairs of a known SNP were excluded.

To identify heritable DNA methylation marks (whether epimutations or mQTLs), we calculated a measure of heritability, $\Delta l$, for each methylation mark, with high values of $\Delta l$ corresponding to Mendelian patterns of inheritance within the families [1]. Because we were interested in causes of familial prostate cancer, we selected only the 1,000 most heritable methylation marks. (Other choices, such as the 2,000 most heritable methylation marks, or all marks with $\Delta l$ above a pre-determined threshold, would be just as statistically valid, but we followed the approach of our previous study [1]). For each of these 1,000 most heritable methylation marks, we calculated the probability that each family member carries a hypothetical genetic variant causing aberrant methylation at the mark, based on M-values and family structure but not ages or affected statuses. We tested these carrier probabilities for association with prostate cancer using Cox proportional hazards survival models. We accounted for multiple testing (for 1,000 tests) using the Bonferroni p-value threshold of 0.05/1000. Risk estimates from the family-based phase are biased by ascertainment so are not presented, though p-values are valid because the test statistic is not affected by ascertainment under the null hypothesis.

The population-based phase was based on unrelated individuals recruited irrespective of family history to the Melbourne Collaborative Cohort Study (MCCS) [7, 8]. Peripheral blood DNA methylation was measured in 869 incident cases (including 430 aggressive cases) and matched controls (matched on year of birth, year of blood draw, country of birth, and sample type) using the HM450 array, as described previously [7, 8]. This data was used to further investigate the marks from the family-based phase that are heritable, associated with prostate cancer risk, and common to the EPIC and HM450 arrays (the two arrays used in the two phases). These marks were tested for association with prostate cancer using conditional logistic regression adjusted for body-mass index, tobacco smoking, alcohol consumption, age at blood draw and estimated blood cell composition. A genome-wide search for mQTLs was also conducted for these methylation marks, using 4,307 unrelated MCCS participants genotyped on the OncoArray-500K BeadChip [3].

# References

1. Joo, J.E., et al., *Heritable DNA methylation marks associated with susceptibility to breast cancer.* Nat Commun, 2018. **9**(1): p. 867.

2. Dugue, P.A., et al., *Heritable methylation marks associated with breast and prostate cancer risk.* Prostate, 2018. **78**(13): p. 962-969.

3. Dugue, P.A., et al., *VTRNA2-1: Genetic Variation, Heritable Methylation and Disease Association.* Int J Mol Sci, 2021. **22**(5).

4. MacInnis, R.J., et al., *Population-based estimate of prostate cancer risk for carriers of the HOXB13 missense mutation G84E.* PLoS One, 2013. **8**(2): p. e54727.

5. Aryee, M.J., et al., *Minfi: a flexible and comprehensive Bioconductor package for the analysis of Infinium DNA methylation microarrays.* Bioinformatics, 2014. **30**(10): p. 1363-9.

6. Fortin, J.P., et al., *Functional normalization of 450k methylation array data improves replication in large cancer studies.* Genome Biol, 2014. **15**(12): p. 503.

7. Milne, R.L., et al., *Cohort Profile: The Melbourne Collaborative Cohort Study (Health 2020).* Int J Epidemiol, 2017. **46**(6): p. 1757-1757i.

8. FitzGerald, L.M., et al., *Genome-Wide Measures of Peripheral Blood Dna Methylation and Prostate Cancer Risk in a Prospective Nested Case-Control Study.* Prostate, 2017. **77**(5): p. 471-478.
